# Lessons learned from 15 years of non‐grades‐based selection for medical school

**DOI:** 10.1111/medu.13462

**Published:** 2017-10-06

**Authors:** Karen M Stegers‐Jager

**Affiliations:** ^1^ Institute of Medical Education Research Rotterdam, Erasmus MC University Medical Centre Rotterdam Rotterdam The Netherlands

## Abstract

**Context:**

Thirty years ago, it was suggested in the Edinburgh Declaration that medical school applicants should be selected not only on academic, but also on non‐academic, attributes. The main rationale behind extending medical school selection procedures with the evaluation of (non‐academic) personal qualities is that this will lead to the selection of students who will perform better as a doctor than those who are selected on the basis of academic measures only. A second rationale is the expectation that this will lead to a representative health workforce as a result of reduced adverse impact. The aims of this paper are (i) to describe what can be learned about the use of selection criteria other than grades from over 15 years of Dutch experience and (ii) to summarise current knowledge on the issue of adverse impact in relation to non‐grades‐based selection.

**Methods:**

A narrative review was undertaken of the (published) evidence that has resulted from non‐grades‐based school‐specific selection procedures in the Netherlands and from recent explorations of the effect of the use of non‐grades‐based selection criteria on student diversity.

**Results:**

The Dutch evidence is grouped into five key themes: the effect of participation in voluntary selection procedures, the assessment of pre‐university extracurricular activities, the use of work samples, Dutch experiences with situational judgement tests and the effects of changing circumstances. This is followed by several lessons learned for medical schools that aim to increase their student diversity.

**Conclusion:**

Over the last 30 years, important steps towards reliable and valid methods for measuring non‐academic abilities have been taken. The current paper describes several lessons that can be learned from the steps taken in the Dutch context. The importance of sharing evidence gathered around the globe and building on this evidence to reach our goal of predicting who will be a good doctor is acknowledged.

## Abstract 2

The author provides several globally applicable lessons learned from over 15 years of experience with non‐grades‐based selection in the Netherlands and describes how non‐grades‐based selection may reduce adverse impact.

## Introduction

According to the 1988 Edinburgh Declaration,^1^ the aim of medical education is ‘to produce doctors who will promote the health of all people’. At that time, that aim was not being realised in many places.^1^ Unfortunately, until today, we have still not realised that aim. In 2006, Betancourt described the role medical schools had to play in increasing the proportion of under‐represented minorities in the health care workforce in order to eliminate the on‐going racial and ethnic disparities.^2^ The promise of improved health care provision, in particular for minority populations, is also one of the arguments for current widening access initiatives.^3^, ^4^ Despite these initiatives, in particular in the USA and the UK, non‐traditional students, such as ethnic minority and first‐generation university students, are still under‐represented in medical schools.^3^, ^5^ Nevertheless, there has been progress, probably because of actions taken by medical schools. One of the actions suggested by the Edinburgh Declaration was to ‘Employ selection methods for medical students which go beyond intellectual ability and academic achievement to include evaluation of personal qualities’.

A first rationale behind extending medical school selection procedures with the evaluation of personal qualities is that this will lead to the selection of students who will perform better as a doctor than those who are selected on the basis of academic measures only. However, what personal qualities should one be looking for? The list of desirable personal qualities is likely to be endless; a 2003 review of the literature revealed up to 87 different qualities relevant to the practice of medicine.^6^ So far, attempts to measure personal qualities in selection have been focused on skills such as ethical decision making, communication and collaboration skills.^4^, ^7^, ^8^ Other attempts have been focused on measuring desirable traits in applicants, such as conscientiousness^9^, ^10^ and empathy.^11^ Recently, there have been calls to also include potential for creativity and innovation as a selection criterion for medical school.^12^ Non‐academic skills are often measured using contextualised instruments, such as the multiple mini‐interview (MMI), consisting of a series of short, structured interviews,^7^, ^13^ or situational judgement tests (SJT), presenting responses to challenging job‐related (or medical school‐related) situations that applicants have to judge for their appropriateness.^14^, ^15^ Current evidence shows that attempts to measure non‐academic skills using the contextualised MMIs^7^ or SJTs^4^, ^8^ are more effective than attempts to measure desirable traits using self‐report questionnaires.^16^, ^17^

A second rationale for the inclusion of criteria other than grades is to ensure a representative health workforce, as this will lead to improved health care provision, in particular for minority populations.^3^, ^4^ As the use of grades has been shown to introduce a significant bias regarding socio‐economic class, especially in the UK,^3^ the promise of the use of non‐grades‐based selection is that this will lower adverse impact on non‐traditional students. Unfortunately, the introduction of non‐grades‐based selection criteria has shown mixed results with respect to student diversity.^3^, ^4^, ^18^, ^19^, ^20^ It was noted that adding measures with reduced adverse impact to existing selection procedures can yield only modest reductions in adverse impacts and sometimes even can have negative effects.^21^

The aims of selecting the best (future) doctors and ensuring a representative health care workforce are further complicated by the following unresolved issues.

The main responsibility of medical schools is to produce competent doctors who will serve societal needs. Therefore, the goal of selection is to identify those who will be successful in medical training and who will ultimately become competent doctors. This in itself already presents a first dilemma for medical schools: should the focus be on selecting the best students or the best doctors? Put differently, which outcomes do we want to predict? This relates to an ongoing debate in selection research: whether or not links to post‐academic outcomes (i.e. performance as a doctor) are necessary to justify the use of a particular selection method.^22^

If we would like to focus on the best doctors, we are confronted with another dilemma: What is the definition of a good doctor? Do we need one type of doctor or different types? A related difficulty is how to find credible measures of performance in future practice that can be related to the personal qualities assessed during selection. Currently, measures used include supervisor ratings^8^, ^23^ and incidence of remedial action^23^ in postgraduate training or results on national licensure examinations,^7^, ^24^, ^25^ with promising outcomes. Nevertheless, it remains extremely difficult to predict at the time of admission to medical school who will be a good doctor many years later.

A focus on selecting the best students raises additional questions. Who will be the best‐performing students depends on the kind of performance you are looking for. Ideally, there is an alignment between admission practices, curriculum (objectives) and assessment practices. This was nicely illustrated by Lievens and colleagues,^26^ who found that a selection tool measuring interpersonal skills only showed incremental validity over cognitively oriented measures for curricula that included interpersonal courses.

Another issue is whether it would be more effective to select students based on personal qualities or skills or to teach and develop these qualities or skills at medical school. Although evidence shows that at least some qualities are trainable,^27^ others argue that specific skills and qualities, next to high academic ability, may need to be present from the start.^17^ One might argue that the aim would be to select applicants with the highest potential to develop the desirable non‐academic skills during medical training. A related question is whether the focus should be on ‘selecting in’ or on ‘selecting out’ on the basis of (unsuitable) personal qualities.^28^

The call for selection procedures that also include the evaluation of personal qualities has led to a divide between so‐called cognitive (or academic or intellectual) and non‐cognitive (or non‐academic or non‐intellectual) tests or criteria. However, non‐cognitive instruments are very likely to also include cognitive components,^29^ and cognitive measures like pre‐university grade point average (pu‐GPA) might also reflect personal qualities such as efficient study strategies and time management.^30^ Additionally, several studies have shown that non‐academic and academic qualities are not independent: success on non‐academic criteria enables success on academic criteria and vice versa.^7^, ^31^, ^32^ Hence it might be more appropriate to speak about ‘broadened admission criteria’,^21^ referring to criteria beyond traditional measures of intellectual ability and pu‐GPA.

A first aim of this paper is to describe what can be learned about the use of selection criteria other than grades from over 15 years of Dutch experiences. A second aim is to summarise current knowledge on the issue of adverse impact in relation to non‐grades‐based selection. Rather than a systematic review of the literature, I conducted a narrative review guided by my own experience with research into non‐grades‐based selection and by the evidence that has resulted from more than 15 years of non‐grades‐based school‐specific selection procedures in the Netherlands. The Dutch (published) evidence is grouped into five key themes: the effect of participation in voluntary selection procedures, the assessment of pre‐university extracurricular activities, the use of work samples, Dutch experiences with situational judgement tests and the effects of changing circumstances.

## Non‐Grades‐Based Selection in The Netherlands

In the Netherlands, since 2000 until very recently, students were selected for medical school either on the basis of a national lottery system that was weighted for pre‐university grades or on medical school‐specific selection procedures. The medical schools themselves could decide on their selection criteria, but were not allowed to use pre‐university grades. The introduction of these school‐specific procedures next to the national lottery system created opportunities for natural experimentation. Additionally, right from the start, medical schools had to consider criteria other than grades. The percentage of students who are admitted via the school‐specific procedures has steadily increased from a maximum of 50% in 2000 to currently 100%. Starting from the academic year 2017–2018, the national lottery system was abolished. With this abolishment Dutch medical schools are now permitted to include pre‐university grades in their school‐specific procedures. The evidence presented below is based on the situation where admission by lottery and by school‐specific selection co‐existed.

### A participation effect?

Until very recently, a unique feature of the Dutch admission system was that medical school applicants could decide whether or not to participate in school‐specific selection procedures. Even students who were initially rejected in one of the voluntary school‐specific selection procedures still had a chance of admission via the lottery system in the same year. This situation offered the unique possibility to study the effects of the school‐specific selection procedures, including the effects of participation.

One of the studies by our group showed a significant lower dropout rate for participants than for non‐participants in our two‐phase selection procedure.^33^ An explanation offered for the difference in dropout was self‐selection instigated by the selection procedure. Because our selection procedure required high levels of both the quality and quantity of extracurricular activities, potential applicants may have decided beforehand not to apply because they would not meet these criteria. An alternative explanation is that participation in the selection procedure is associated with a higher motivation to become a medical doctor, reflected in grasping this additional chance to enter medical school.^33^ Although this explanation has also been suggested by others,^31^ a recent multi‐site study failed to confirm the expected higher motivation for participants than for non‐participants in school‐specific selection procedures.^34^ Given the time‐consuming nature of most Dutch selection procedures,^31^, ^35^ it was not surprising that participants were found to score higher on conscientiousness than non‐participants.^36^

Other studies, from Denmark and the Netherlands, also revealed this so‐called participation effect: non‐participants were at higher risk of dropout,^37^ study delay^31^ and lower grades^31^ than participants. Apparently, in predicting academic performance, *participation* in a time‐consuming voluntary selection procedure is more important than *acceptance*.^31^, ^33^, ^37^ Unfortunately, the earlier reported participation effects with respect to study delay and lower grades were not consistently confirmed in a follow‐up multi‐site study.^38^ Hence, the conclusion was that participation effects seem to be mediated by institutional differences in curricula and in selection procedures.^38^

### Assessing pre‐university extracurricular activities

Several Dutch medical schools, including our own, have operationalised non‐academic skills by examining applicants’ extracurricular activities during pre‐university education (puECAs).^31^, ^33^ This method has the advantage of an increased authenticity, because it reflects students’ development over the last couple of years instead of being based on a ‘single’ test administration. In our school, for example, applicants are assessed on the quality and quantity of their extracurricular activities 2.5 years before application, based on verifiable information provided on the application form. Extracurricular activities include paid and unpaid jobs in health care, experience in management and organisation, or those that show special talents in domains such as music, science or sports (see Urlings‐Strop et al. 2009^35^ for a more extensive description of our selection procedure).

The first studies by our group showed that students selected by our two‐phase selection procedure, including the assessment of puECAs, had a 2.6 times lower relative risk of dropout than students admitted by lottery^35^ and received significantly higher mean grades on their first five clerkships.^39^ A Danish study also showed encouraging results in preventing dropout by using puECAs as part of their attributes‐based selection procedure.^37^ Another Dutch study found that their multifaceted selection procedure, including puECAs, was particularly efficient in identifying applicants with suitable ‘non‐academic’ skills, such as professionalism.^31^

A follow‐up study from our group focusing on the relative contribution of the different facets of the selection procedure revealed that whereas both the non‐academic (i.e. puECAs) and academic (i.e. cognitive tests) selection criteria were associated with a lower chance of dropout during medical school, the better clinical performance of selected students was almost exclusively related to the non‐academic selection criteria.^33^ In a subsequent study we compared students selected (exclusively) on non‐academic criteria and students selected (exclusively) on academic criteria with lottery‐admitted students. Contrary to our expectations, students selected on non‐academic criteria did not outperform lottery‐admitted students in pre‐clinical training, whereas those selected on academic criteria did.^40^ This led us to conclude that apparently the use of non‐academic selection criteria is not sufficient to select the best academically performing students, probably because a minimum academic level is required to succeed in medical school. It should be noted, however, that this study did not look at differences in clinical performance between the three groups. In our most recent study we found that students selected on the basis of their puECAs persisted in their ECAs during medical school and that this persistent participation was related to better clinical performance, which further supports the inclusion of puECAs in the selection procedure.^41^

It is good to note that although puECAs can be seen as a form of personal statements,^17^ their assessment, at least in the Dutch medical schools, is different from analysing the content of free response personal statements, as for example described by Ferguson et al.^42^ The use of a highly structured application form in combination with a highly structured rating form provides a high degree of structure to the raters’ judgements. This may positively affect the criterion‐related validity, in the same way as job interviews are positively affected by the degree of structure.^43^ This may explain our positive findings compared with those on personal statements recently summarised in a review by Patterson et al.^17^ Consequently, puECAs may be used successfully as a selection tool provided that the judgement process is sufficiently structured.

### Work samples

The use of work samples, well known in personnel selection, refers to a situation where a sample of future expected behaviour is taken as a predictor of future success in the job.^44^ The focus on *samples* instead of *signs* (i.e. distinguishable constructs, traits or skills) distinguishes them from traditional selection methods used in medical education.^45^ The reasoning behind this approach is that, following the notion of behavioural consistency,^44^ the more the predictor and criterion are alike, the higher the predictive validity will be.^21^ As early performance is the best predictor of future (medical school) performance,^46^, ^47^, ^48^ developing an admission test similar to the first course in the university programme sounds promising.

In the context of medical school selection, Lievens and Coetsier^45^ found a correlation of 0.19 for two ‘miniaturised’ work samples (i.e. a videotaped lecture and reading a medical text) with first‐year GPA and concluded that these work samples could significantly better predict first‐year GPA than cognitive ability tests. In a more recent study De Visser et al.^49^ showed additional value of their curriculum sample selection procedure compared with pu‐GPA, in particular for the lower pu‐GPA categories. Other recent Dutch studies also showed positive results for the use of curriculum samples for the selection of undergraduate psychology applicants.^46^, ^50^, ^51^ In all the above studies, applicants were required to watch a videotaped lecture or study introductory domain‐specific material independently at home, followed by an examination at the university, just like the reality at university. Positive findings for this efficient method for large groups^49^ are explained by the high representativeness of the sampled behaviour for the educational programme it was designed for.^51^ Actually, the selection procedures applied in Amsterdam, Rotterdam and Groningen also contain ‘lecture tests’.^34^, ^35^

The question of course is, what exactly is measured by sample‐based tests, and whether such tests should be considered academic or non‐academic measures. De Visser et al.^49^ describe their curriculum sample approach as selection on an academic basis, although it could also be argued that the sample‐based approaches described above measure a mixture of cognitive ability, motivation, time spent studying, and tactic knowledge.^21^ At least they measure applicants’ performance on authentic tasks that represent behaviour that is required of them during the pre‐clinical years of medical school, which might explain the additional value of the tests over pu‐GPA. It is important to note that the examples mentioned here are focused on selecting the best students rather than the best doctors.

### Situational judgement tests

An example of a non‐academic measure that is increasingly used for selection into medical school is the situational judgement test (SJT). As mentioned above, SJTs present applicants with several situations that they may encounter during the job (or at medical school), followed by a number of possible responses to that situation, of which they have to judge the appropriateness. SJTs have consistently been shown to be a reliable and valid method to assess various personal qualities that are important to medical doctors, such as integrity, empathy and teamwork.^17^ Thanks to these positive findings, Dutch medical schools have started using (or piloting) SJTs as part of their school‐specific selection procedures.

As a first example, the medical school in Groningen used an SJT to measure professionalism in various medical contexts and found that both previous academic experience and a good fit between applicants’ vocational interests and SJT scenarios were related to better SJT performance.^52^ As suggested by the researchers, SJTs could hence be used to select appropriate candidates for areas of health care that are in need of more professionals.^52^

At Erasmus MC Medical School we first pilot‐tested an integrity‐based SJT developed in the UK,^53^ which was translated into Dutch. We learned two important lessons from this endeavour. First, one should be cautious in using SJTs developed abroad and tailored to the specific contexts. There were substantial differences between the original UK scoring key and the scoring key we developed using Dutch experts. Additionally, not all items were considered relevant or appropriate to the Dutch context by our participants. Finally, using the same scoring method as in the UK context revealed a low internal consistency reliability. Following on from these findings we decided to explore 28 (!) alternative scoring methods.^54^ This revealed a second important lesson: the increased use of SJTs in medical school selection must be accompanied by a thorough examination of the scoring method to be used. We found that the applied scoring method has a strong influence on the internal consistency reliability and adverse impact of an SJT score.^54^ Currently, we are in the process of pilot‐testing an in‐house‐developed integrity‐based SJT tailored to our own context. Preliminary findings suggest that scoring an SJT based on the ability to recognise what one should not do as opposed to the ability to recognise what one should do in a challenging situation strengthens the convergent validity of that SJT (unpublished results).

Unexpectedly, our intention to include integrity‐related aspects in our selection procedure has caused some controversy in the Netherlands. Adversaries doubt whether it is fair to judge young adults on integrity‐related issues and propose that these behaviours are teachable. Therefore, it might be better to ‘red‐flag’ applicants with low scores on integrity‐related measures and offer them (additional) training instead of rejecting them. Others even go so far as to say that it is unethical to report applicants’ scores on integrity‐related measures to the client (the medical school), and state that such scores should only be reported to the applicants themselves, who then can decide whether or not to continue with their application.^55^, ^56^ This is related to the remark made by Patterson et al.^17^ that personality assessments should not be used as a selection method on their own, but rather as input for selection interviews.

However, our integrity‐based SJT is intended to measure knowledge and skills (how one should act) rather than personality traits (how you would act). In our opinion, a certain ability to identify appropriate and inappropriate reactions to challenging situations is essential for our future medical doctors. We also expect applicants passing our SJT to be more responsive to training in professional behaviour during medical education than applicants failing the SJT.

### Effect of changing circumstances

So far, studies from the Netherlands have reported conflicting evidence, with some reporting positive effects (i.e. better performance for selected students compared with lottery‐admitted students) and some, especially more recent studies, reporting little or no effect. A possible explanation is the increased proportion of students admitted through selection (from 50% to 100%). Moreover, the number of medical schools using school‐specific selection procedures increased from only a few to currently all. The effect of increasing the percentage of students admitted by selection on the added value of selection to lottery can be explained using the Taylor‐Russell model. This model calculates success ratios (equals the proportion of admitted students who will be successful in medical school and as a doctor) as a function of the base rate, selection ratio and predictive validity of the test(s) used for selection (see also Niessen & Meijer 2016^57^). When the selection ratio (equals percentage of selected applicants) increases without changes in the base rate (equals percentage of suitable applicants), the additional value of selection decreases. Data from the time when all medical students were admitted via lottery show that for all Dutch medical schools the graduation rate was about 85%,^58^ suggesting a high base rate. A main reason for this high base rate is the fact that medical school applicants in the Netherlands form a very homogenous group.^40^ Only students who completed the highest level of secondary school (equals pre‐university level) and took the six subjects required for medical school are allowed to apply (less than 10% of all high school students).

Another explanation might be that applicants’ behaviour or the rationale behind this behaviour change when admission criteria become known. For example, considering our own context, it might be that, following Higgins’ regulatory focus theory,^59^, ^60^ when the requirement of puECA participation for admission became more transparent, applicants may have chosen to participate in puECAs because they felt they ‘had to’ do this to have a chance to get into medical school, and not because they ‘wanted to’. Exploring the regulatory focus of currently admitted students and relations with performance may be an interesting area for further research. The more general lesson from this is that medical schools should be aware of the possible effects of selection procedures on applicants’ behaviour. This is especially important as applicants have been shown to base their choice of a medical school more often on the selection procedure than on the curriculum.^61^

## Adverse Impact

As noted previously, the introduction of broader admission criteria has shown mixed results with respect to student diversity.^3^, ^4^, ^18^, ^19^, ^20^ Nevertheless, recent efforts have led to several lessons for medical schools that aim to increase the diversity of their student population.

First, the effect of using an instrument with reduced or no adverse impact on student diversity depends on the weight given to the instrument and the selection ratio. This was illustrated by Lievens et al.,^4^ who found that the positive effect on the representation of lower SES candidates was stronger when the UKCAT SJT had more weight (i.e. 50%) and the selection ratio was more stringent (i.e. selecting the top 15%). Likewise, we have advocated previously in this journal that medical schools should explore the impacts of different weightings of non‐academic and academic criteria on student diversity.^32^

Second, different selection criteria should be used concurrently.^32^ This prevents the diversity‐limiting effect of considering personal qualities only once grade requirements have been met.^20^, ^62^ Additionally, to increase diversity it might be better to allow applicants to make up for lower scores on some criteria with high scores on others (compensatory) instead of expecting them to score highly on all selection criteria (non‐compensatory).^32^ Currently, at the Erasmus MC medical school a compensatory system is used. Scores on the three different elements of our selection procedure (a mixture of academic and non‐academic criteria) are transformed into z‐scores, and applicants are ranked according to their average z‐score.

Third, including non‐academic criteria in the selection procedures does help to increase the diversity of medical student populations.^32^ For example, our non‐academic selection criterion (i.e. the use of extracurricular activities) did not show an adverse impact with respect to ethnicity or social background.^32^ However, as advocated previously in this paper, selection procedures should not rely on non‐academic criteria alone, but should also include academic criteria.^33^, ^40^

Fourth, one should be aware of self‐selection (i.e. resulting in not applying) in non‐traditional applicants. As we have argued previously,^32^ it cannot be ruled out that self‐selection instigated by selection procedures is stronger in applicants from non‐traditional backgrounds. Although the percentage of non‐Western minority applicants in our study by far exceeded the percentage of non‐Western pre‐university graduates in the Netherlands (27% versus 8%) and the percentage of first‐generation students was higher among applicants than among current students (including those admitted by lottery), we still do not have any data about those who decided not to apply. A recent qualitative study, albeit small, from Amsterdam suggests that selection procedures currently applied in the Netherlands may discourage students without a ‘medical network’ from applying to medical school, therefore leading to inequalities with respect to socio‐demographic background.^63^ An interesting area of research would be whether certain types of (non‐academic) selection instruments are less discouraging for non‐traditional applicants, for example because of higher face validity.

Finally, the choice of selection procedure may also influence the gender composition of the student population. Aptitude tests were found to favour men (i.e. male applicants had a higher chance of being admitted based on aptitude tests than female applicants),^64^ whereas instruments measuring non‐academic qualities such as SJTs seem to favour female students.^4^ In the Netherlands, where currently two‐thirds of the students are female, female students were more likely to be admitted through a multifaceted selection procedure that included reflection assignments and MMI‐like interviews^31^ or SJT scenarios,^52^ whereas the use of puECAs as selection criterion did not show gender‐related differences.^32^

## Conclusion

Summing up, although there are still multiple issues to be solved regarding the use of selection criteria other than grades, several globally applicable lessons can be learned from the Dutch ‘natural experiments’. First, there appears to be a participation effect, in that students who participated in voluntary selection procedures performed better at medical school than non‐participants. However, the magnitude of the effect depends on the curriculum and the selection procedure of the medical school. Second, using pre‐university extracurricular activities for selection to medical school seems to lead to lower dropout rates and better pre‐clinical and clinical performance, provided that the judgement process is sufficiently structured. Third, the use of curriculum samples is another promising selection method, particularly when the aim is to select those students who will perform best in your curriculum. Fourth, contextualised instruments such as SJTs should really be tailored to the local context and should be accompanied by a careful examination of the scoring method to be used. Fifth, one should be aware of the possibility of controversy when including the assessment of (certain) personal qualities in selection procedures. Sixth, changing circumstances, such as higher selection ratios or applicants’ adapting behaviour to meet the selection criteria, may decrease the predictive validity of selection methods. Finally, with respect to adverse impact the main recommendation for medical schools would be to carefully think about a combination and weighting of academic as well as non‐academic selection instruments that would fit both the needs of validity and of diversity.^4^, ^17^, ^32^, ^65^

Issues regarding reliability, validity and fairness of selection procedures have led to recent calls from the UK, the US and the Netherlands to replace medical school selection with lotteries.^66^, ^67^, ^68^ However, my opinion is that, as long as we prevent our selection procedures from resembling expensive lotteries^69^ by ensuring their reliability and validity while also taking the diversity amongst applicants into account, it is preferable to consider perceptions of applicants who favour methods that put them ‘in control’^70^ as well. Over the last 30 years, important steps towards reliable and valid selection methods for measuring non‐academic abilities have been taken, several of them in the Dutch context as described in the current paper. Our task of predicting who will be a good doctor will remain extremely complex and there is much that we still have to understand. While acknowledging this, it is important to share and build on the evidence that is gathered around the globe in order to get a little closer to our goal.

## Contributors

the author agrees to be accountable for all aspects of the work in ensuring that questions related to the accuracy or integrity of any part of the work are appropriately investigated and resolved.

## Funding

none.

## Conflicts of interest

none.

## Ethical approval

no ethical approval was sought for this project.
